# Altered Active Zones, Vesicle Pools, Nerve Terminal Conductivity, and Morphology during Experimental MuSK Myasthenia Gravis

**DOI:** 10.1371/journal.pone.0110571

**Published:** 2014-12-01

**Authors:** Vishwendra Patel, Anne Oh, Antanina Voit, Lester G. Sultatos, Gopal J. Babu, Brenda A. Wilson, Mengfei Ho, Joseph J. McArdle

**Affiliations:** 1 Department of Pharmacology and Physiology, New Jersey Medical School-Rutgers University, Newark, New Jersey, United States of America; 2 Department Cell Biology and Molecular Medicine, New Jersey Medical School-Rutgers University, Newark, New Jersey, United States of America; 3 Department of Microbiology, University of Illinois, Urbana-Champaign, Illinois, United States of America; University of Sydney, Australia

## Abstract

Recent studies demonstrate reduced motor-nerve function during autoimmune muscle-specific tyrosine kinase (MuSK) myasthenia gravis (MG). To further understand the basis of motor-nerve dysfunction during MuSK-MG, we immunized female C57/B6 mice with purified rat MuSK ectodomain. Nerve-muscle preparations were dissected and neuromuscular junctions (NMJs) studied electrophysiologically, morphologically, and biochemically. While all mice produced antibodies to MuSK, only 40% developed respiratory muscle weakness. In vitro study of respiratory nerve-muscle preparations isolated from these affected mice revealed that 78% of NMJs produced endplate currents (EPCs) with significantly reduced quantal content, although potentiation and depression at 50 Hz remained qualitatively normal. EPC and mEPC amplitude variability indicated significantly reduced number of vesicle-release sites (active zones) and reduced probability of vesicle release. The readily releasable vesicle pool size and the frequency of large amplitude mEPCs also declined. The remaining NMJs had intermittent (4%) or complete (18%) failure of neurotransmitter release in response to 50 Hz nerve stimulation, presumably due to blocked action potential entry into the nerve terminal, which may arise from nerve terminal swelling and thinning. Since MuSK-MG-affected muscles do not express the AChR γ subunit, the observed prolongation of EPC decay time was not due to inactivity-induced expression of embryonic acetylcholine receptor, but rather to reduced catalytic activity of acetylcholinesterase. Muscle protein levels of MuSK did not change. These findings provide novel insight into the pathophysiology of autoimmune MuSK-MG.

## Introduction

Autoimmune MG is a disorder that reduces the safety factor of neuromuscular transmission [1]–[4]. The endplate acetylcholine receptor (AChR) was the only identified target for autoimmune MG until 2001, when Hoch and colleagues reported antibodies to MuSK in 70% of AChR-seronegative MG patients [5]. Subsequent studies, reported that 40 to 60% of AChR-seronegative patients had MuSK antibodies [6]–[8].

MuSK-MG is prevalent in females and has a low incidence of complete stable remission. Bulbar and respiratory muscles are severely affected so that respiratory insufficiency is frequently observed in MuSK-MG patients [9],[10]. Current MuSK-MG therapies are limited. Plasmapheresis and intravenous immunoglobulin relieves acute respiratory distress [10]. Although immune suppression with Rituximab improves symptoms [11]–[13], not all patients respond and those that do often become refractory [14]. While the benefit of thymectomy is unclear [6],[15], anti-AChE drugs do not improve and may even worsen MuSK-MG weakness [15]–[18]. The non-responsiveness to AChE inhibitors, fluctuation of symptoms, and sparing of limb muscle hinders early diagnosis of MuSK-MG [12]. Furthermore, long-term non-synaptic effects arising from reduced neuromuscular activity [19] may negatively impact the effectiveness of therapies that selectively target the NMJ. Therefore, improved understanding of the overall pathophysiology will improve MuSK-MG diagnosis and treatment as in the case of AChR-MG [20].

MuSK plays an essential role in the overall development and maintenance of the NMJ, including clustering of the AChR [21]–[28]. For example, MuSK regulates expression and activity of acetylcholinesterase (AChE) at the NMJ [29],[30]. MuSK antibodies may disrupt this regulatory influence to produce the unresponsive or deleterious response of MuSK-MG patients to anti-AChE drugs [15],[18]. In animal models of MG, anti-MuSK antibodies disrupted pre- and post-synaptic function at the NMJ and revealed a significant loss of AChRs at the motor endplate [31]–[36]. However, biopsies of weakened muscle obtained from MuSK-MG patients do not reveal a significant decline of endplate AChR density [30],[37],[38], although electrophysiological studies of similar biopsies reported decreased endplate potential (EPP) and miniature endplate potential (mEPP) responses [38].

The process of synaptic homeostasis [39],[40], via retrograde signaling, enables motor nerves to compensate for post-synaptic receptor loss [41] or endplate AChR loss during AChR-MG [42] by increasing neurotransmitter release. Studies of MuSK-MG animal models suggest that neurotransmitter release declines [32],[34],[43],[44]. This suggests that the homeostatic process may be inactivated. Furthermore, MuSK antibodies may alter retrograde signaling from the muscle to nerve by disrupting MuSK-Lrp4 binding interaction [45]. Therefore, we tested the hypothesis that motor-nerve dysfunction, in combination with loss of endplate AChRs, contributes to the reduced safety factor for neuromuscular transmission during MuSK-MG. This hypothesis raises fundamental questions that are clinically relevant since altered motor-nerve function would complicate therapy. Therefore, it is essential to understand the impact of MuSK-MG on motor nerve functions.

This goal is made possible by active [31]–[33],[46]–[48] as well as passive [33]–[36],[45],[49] immunization models of MuSK-MG [50]. Herein, we immunized mice with rat MuSK ectodomain and evaluated functional, morphological, and biochemical properties of NMJs in respiratory muscles of mice exhibiting MuSK-MG. Our data suggest that the following presynaptic changes contribute to reduced neuromuscular transmission during MuSK-MG: 1) reduced number of active zones, 2) reduced probability of quantal release, 3) reduced number of quanta, and 4) altered nerve terminal conductivity and morphology. These findings provide novel insight into the pathophysiology of MuSK-MG and suggest possible targets for therapeutic intervention.

## Methods

### Cloning and purification of MuSK (22-212) (MuSK)

A DNA vector derived from pRK5 encoding the rat MuSK gene, kindly provided by Dr. Veit Witzemann [26], served as a PCR template to generate a MuSK protein fragment comprised of residues 22-212 [51] using the primers MuSK-kpn-f: 5′-CTGCAGCTGGTACCGCGTGGCAGCGAGAAACTTCCAAAAGCCCCTGTCATC-3′ and MuSK-hind-r: 5′-CAGCCAAGCTTCTATTATGCAAAAACCTCCACTTCCAGCTTCA-3′. After restriction digestion, the fragment was ligated into the KpnI/HindIII site of pGE-GFP vector [52] to obtain pGEpi-MuSK(22-212). This recombinant plasmid was transformed into *E. coli* Rosetta cells for protein expression. The cell-free extract after centrifugation at 14,500×*g* was partially purified through a Nickel-chelation column. The imidazole eluate showed a mixture of multiple species. Heating the sample in the presence of beta-mercaptoethanol (BME) resulted in irreversible precipitates. The imidazole eluate was loaded onto a 5-mL HiTrap ANX-column (GE Health) and developed on FPLC in 50 mM Tris buffer (pH 7.5) containing 2.5% glycerol and 0.1% BME with a 0.1 M NaCl gradient. The fractions containing small molecular weight product corresponding to the expected monomer (26.2 KDa) were pooled and enriched with an additional FPLC purification on an ANX-column. Protein concentration was determined by Bradford assay and results confirmed by Western blot using commercially available antibody corresponding to extracellular domain of MuSK amino (residues 24-209) (Abcam, USA)

### Active immunization with MuSK

All animal procedures were approved by the New Jersey Medical School-Rutgers University IACUC. Female 8-week-old C57/B6 mice were obtained from Harlan Laboratories (USA). All mice were lightly anesthetized with isoflurane prior to subcutaneous injections. 68 experimental mice were injected with 50∶50 mixture of 40 µg of purified rat MuSK ectodomain in phosphate buffered saline (PBS) and complete Freund's adjuvant (CFA) (Difco, Michigan USA); 32 control mice were injected with PBS and CFA without MuSK. Three MuSK injections were made at 0, 4, and 8 weeks. Mice tested on the rotarod were injected in the base of the tail and flank regions to avoid running difficulties associated with footpad injections. None of the control or experimental mice died because of the injection procedure. Thus, MuSK-immunized mice did not reach the end stage of the disease during the time course of our study.

### Rotarod analysis

Mice were first trained for 5 days to run on a horizontal rotating rod (Rotamex, Columbus Instruments, Columbus, OH). During the initial 2 days mice were acclimatized to run at a fixed speed of 10 RPM for a maximum of 10 minutes twice a day. Training was then continued on days 3, 4, and 5, according to the test protocol, where the rate of rotation was gradually increased in increments of 5 RPM until a maximum limit of 20 RPM was reached or the mouse fell from the rod onto a cushioned surface. To obtain a muscle-performance score, mice were subjected to 3 trials on 2 days of each week. The time to fall from the rotating rod was averaged for each set of 3 trials and recorded for each time point after beginning the MuSK injections.

### Grip strength

To measure grip strength, mice holding on to a metal ring with their fore paws were gradually pulled away horizontally. The metal ring was connected to a force transducer (Grass Technologies, West Warwick, RI) connected to a Digidata1440A (Axon Instruments, Molecular Devices, Sunnyvale, CA). pCLAMP 10 software (Axon Instruments) recorded and analyzed the force data. Five trials were recorded. The average of the 3 largest force values was measured at various times after beginning MuSK immunization.

### Plethysmography

Head-out, whole-body plethysmography was performed to assess respiratory function. Briefly, mice were anesthetized with ketamine-xylazine and placed in a chamber with their heads protruding from the chamber. A latex film sealed the neck region so that the body was in an airtight chamber while the mouse breathed outside air. The diaphragm of a small chamber in series with the plethysmograph chamber was attached to a force transducer to detect pressure changes arising from chest-wall expansion and contraction. Transducer signals were amplified, digitized (Digidata 1440A), and recorded with pCLAMP 10 software. The transducer response to a known volume of air enabled calibration of the plethysmograph.

### Muscle mechanics

Mice were anesthetized with isoflurane and sacrificed by cervical dislocation. Phrenic nerve hemidiaphragm preparations were excised quickly and placed into oxygenated (95% O_2_, 5% CO_2_) Ringer's solution (pH 7.4, containing 135 mM NaCl, 5 mM KCl, 1 mM MgCl_2_, 2 mM CaCl_2_, 1 mM Na_2_HPO_4_, 15 mM NaHCO_3_, and 5.5 mM glucose) at room temperature. One tendon of the hemidiaphragm was attached to a hook at the bottom of a Rodnotti chamber and the other tendon was attached to a Grass force transducer. Prior to data acquisition, the hemidiaphragm was stretched to optimal length for maximal force production and was equilibrated for 15 minutes at 0.5-Hz phrenic nerve stimulation. For data acquisition, the phrenic nerve was stimulated sequentially at 2, 20, 50, and 70 Hz with supramaximal stimuli. Transducer signals were amplified, digitized (Digidata 1440A), and recorded with pCLAMP 10 software for off-line analyses.

### Electrophysiology

The triangularis sterni (TS) nerve-muscle preparation [53] was dissected from isoflurane-anesthetized mice and placed in a chamber containing HEPES-Ringer (HR) solution (135 mM NaCl, 5 mM KCl, 2 mM CaCl_2_, 1 mM MgCl_2_, 5.5 mM dextrose, and 5 mM HEPES, pH 7.3–7.4). Preparations were incubated in HR solution containing 0.75 µM μ-conotoxin GIIIB for 30–45 minutes to inhibit muscle contraction in response to nerve stimulation. Two-electrode voltage clamp technique was used to record the spontaneous miniature endplate currents (mEPCs) and endplate currents (EPCs) produced in response to nerve stimulation; a minimum of 80% of endplate potential amplitude was voltage-clamped. pCLAMP 10 software was used for data acquisition and analysis. Quantal content was estimated directly as the ratio of the mean EPC amplitude to the mean mEPC amplitude. In addition, EPCs during trains of high frequency stimuli at 20, 50, and 70 Hz were recorded. For analysis of neurotransmitter release during these trains, the amplitude or quantal content of each EPC was presented as a percentage of the first EPC in the train. A total of 41 EPCs were produced at each frequency. Potentiation, depression, and steady state release of neurotransmitter was analyzed for each train. When high frequency stimulation was applied to preparations, quantal content was estimated by dividing the EPC amplitude of each pulse in the steady state portion of the stimulus train by the average of the mEPC amplitude obtained during 1-Hz stimulation of the same NMJ. This approach was selected because of the difficulty in distinguishing mEPCs during short stimulus train periods. To estimate total functional pool size, the nerve was stimulated at 50 Hz for 2 minutes or less, if EPC amplitudes declined to that of mEPCs.

The number of release sites (N) was estimated from the variability of EPC and mEPC amplitudes recorded at 1-Hz nerve stimulation. N for each NMJ was calculated as 
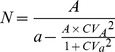
(1)


Where: A  =  mean EPC amplitude; a  =  mean mEPC amplitude; CV_A_  =  coefficient of variation of EPC amplitude; and CV_a_  =  coefficient of variation of mEPC amplitude [54]. The probability of vesicle release (P) for each NMJ was calculated from equation 2, where Q  =  Mean quantal content.
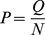
(2)


### Nerve terminal current recording

TS preparations were stained with 30 nM rhodamine conjugated α-bungarotoxin in HEPES Ringer solution for 15 minutes at room temperature. Stained motor endplates were visualized with an upright fluorescence microscope (BX61WI, Olympus America Inc.), so as to focally position a 1-µm tip diameter extracellular recording electrode containing physiological saline.

### AChE activity

Hemidiaphragm muscles were dissected, weighed, and homogenized in 9 volumes of 100 mM sodium phosphate buffer (pH 7.4). Production of thiocholine in the Ellman reaction was used to measure AChE activity [55]. Briefly, a total of 1 ml of reaction volume containing 0.44 mM acetylthiocholine and 0.1 mM 5,5′-dithio-*bis*(2-nitrobenzoic acid) was placed in a cuvette. The reaction was initiated by addition of 50 µl of muscle homogenate, and the increase in optical density at 412 nm (due to thiocholine production) was followed for 10 min in a Shimadzu UV2550 spectrophotometer (Kyoto, Japan). The slopes of optical density curves were determined by linear regression (Sigmaplot 8, Systat Software, Chicago, IL).

### Confocal imaging

TS muscles were fixed in 4% formaldehyde for 15 min at room temperature and washed with 100 mM glycine for 2 hrs, incubated with 60 nM rhodamine conjugated α-bungarotoxin for 1 hr, permeabilized with methanol at −20°C for 10 minutes, and blocked with a solution of 2% BSA and 0.3%Triton-X100 in PBS for 1 hr. The muscles were then incubated with primary antibodies against either neurofilament (Abcam, USA) and synaptophysin (Abcam, USA) or ELKs (Sigma-Aldrich, USA) over night, followed by secondary antibodies (Sigma-Aldrich, USA) for 4–5 hrs. Muscles were washed 3 to 4 times between each step with PBS. The muscles were then mounted on slides in Vecta Shield mounting medium and visualized using an inverted confocal microscope (A1R-A1, Nikon Instruments, Inc.). Images for ‘en face’ NMJs were captured and analyzed using NIS element software (Nikon Instruments, Inc.).

### Immunoblot

For dot-blot analyses, purified MuSK protein was diluted with PBS to make solutions of 1∶10, 1∶100, and 1∶1000 concentration, and 2 µl of each dilution was applied directly to nitrocellulose paper, which was then incubated overnight with a 1∶500 dilution of serum sample from mice injected with MuSK or control vehicle. The nitrocellulose paper was then blocked with 2% BSA for 2 hrs, incubated with anti-mouse HRP-labeled secondary antibody, and analyzed with an Amersham ECL Prime Western blotting detection system (GE Health Care). For Western blot analysis primary antibodies against MuSK (Abcam, USA) and the AChR γ subunit (Santa Cruz Biotechnology, USA) were used, according to manufacturers' instructions.

### Statistics

Data for all figures were expressed as the mean ± S.E.M. Student's *t* test or one-way ANOVA with Tukey *post hoc* test were used to evaluate the statistical significance of population means. The symbols *, **, or *** denote *p*<0.05, *p*<0.001, or *p*<0.0001, respectively.

## Results

### MuSK-induced MG in mice

**Figure 1:**
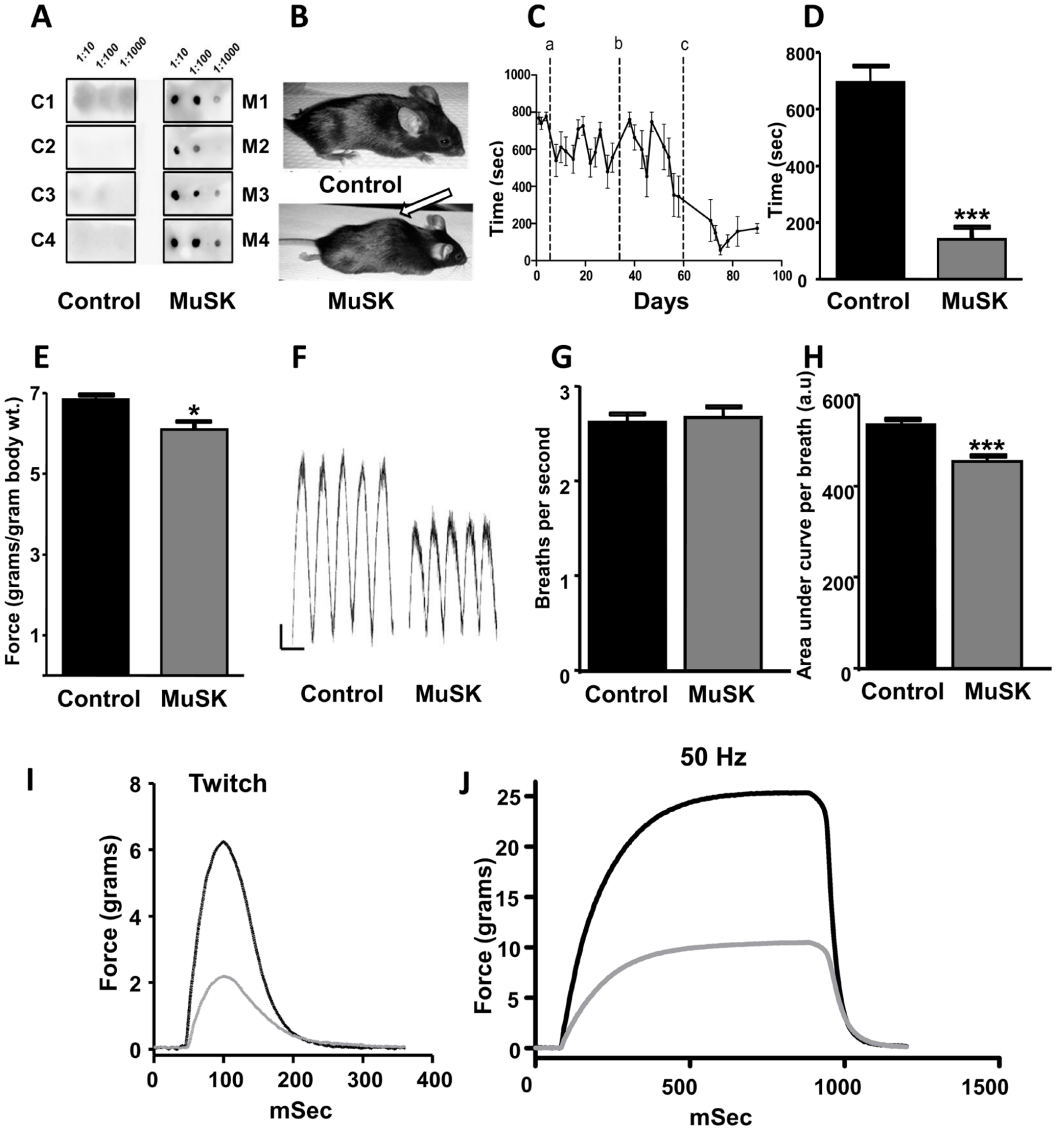
Immunized mice produce MuSK antibodies and have reduced skeletal muscle function. (A) Dot blot analysis detects anti-MuSK antibodies in serum of 4 MuSK-injected (M1-M4), but not vehicle-injected control (C1-C4) mice. Sera were tested against 1∶10, 1∶100, and 1∶1000 dilutions of purified MuSK. (B) Representative images of mice after 3 injections with vehicle (Control) or MuSK. Approximately 20% of MuSK-immunized mice develop kyphosis (arrow) due to weakness of back and neck muscles. Average time on rod rotating at 20 rpm for mice 0–3 days before and at various days after MuSK injections. Dotted lines a, b, and c indicate the times of MuSK injections and data points are shown as the mean + SEM for 6 to 12 mice. (D) Summary of walk time on the rotating rod for 12 vehicle-injected controls and 6 mice injected 3 times with MuSK. (E) Forelimb grip strength normalized to the body mass of 8 controls and 10 MuSK-injected mice. (F) Representative head-out plethysmography records of mice injected thrice with either vehicle (Control) or MuSK. Calibrations: time, 500 mSec, y-axis arbitrary units. (G, H) Respiration rates for 11 controls and 8 MuSK-injected mice, in breaths per second (G) or area under curve of each breathing cycle (H). Representative twitch (2 Hz) (H) and tetanic (J) responses of phrenic-nerve diaphragm muscle preparations from control (black trace) and MuSK-MG-affected (grey trace) mice. All data bars are shown as the mean + SEM; *** denotes P<0.0001, * denotes P<0.05.

We injected 68 8-week-old, female C57/B6 mice with 40 µg of purified, recombinant rat MuSK ectodomain every 4 weeks for a total of 3 injections. None of the MuSK-immunized mice died of myasthenia-related symptoms. While all MuSK-immunized mice produced antibodies, only 20% developed kyphosis as a sign of severe neck muscle weakness. However, generalized skeletal muscle weakness increased gradually for the entire MuSK-immunized cohort, as indicated by reduction of walk time on a rotating rod, grip strength, and respiratory activity (Figure 1 A to H). Because 60 to 70% of MuSK-MG patients suffer respiratory muscle weakness [16],[56],[57], we recorded twitch and tetanic force responses of isolated hemidiaphragm muscles to phrenic nerve stimulation (Figure 1 I and J). As reported for other muscles [32],[34],[58], diaphragm muscle force was 80% or less of control values in approximately 40% (22 of 54 mice) of the MuSK-immunized mice, with the remaining MuSK-immunized mice showing normal diaphragm muscle force generation and quantal content (Figure S1 A and B). The 40% of MuSK ectodomain-immunized mice with weakened diaphragms are referred to as MuSK-MG-affected.

### Decrease of AChR density, neurotransmitter release, and EPC failures at NMJs in MuSK-MG-affected mice

**Figure 2:**
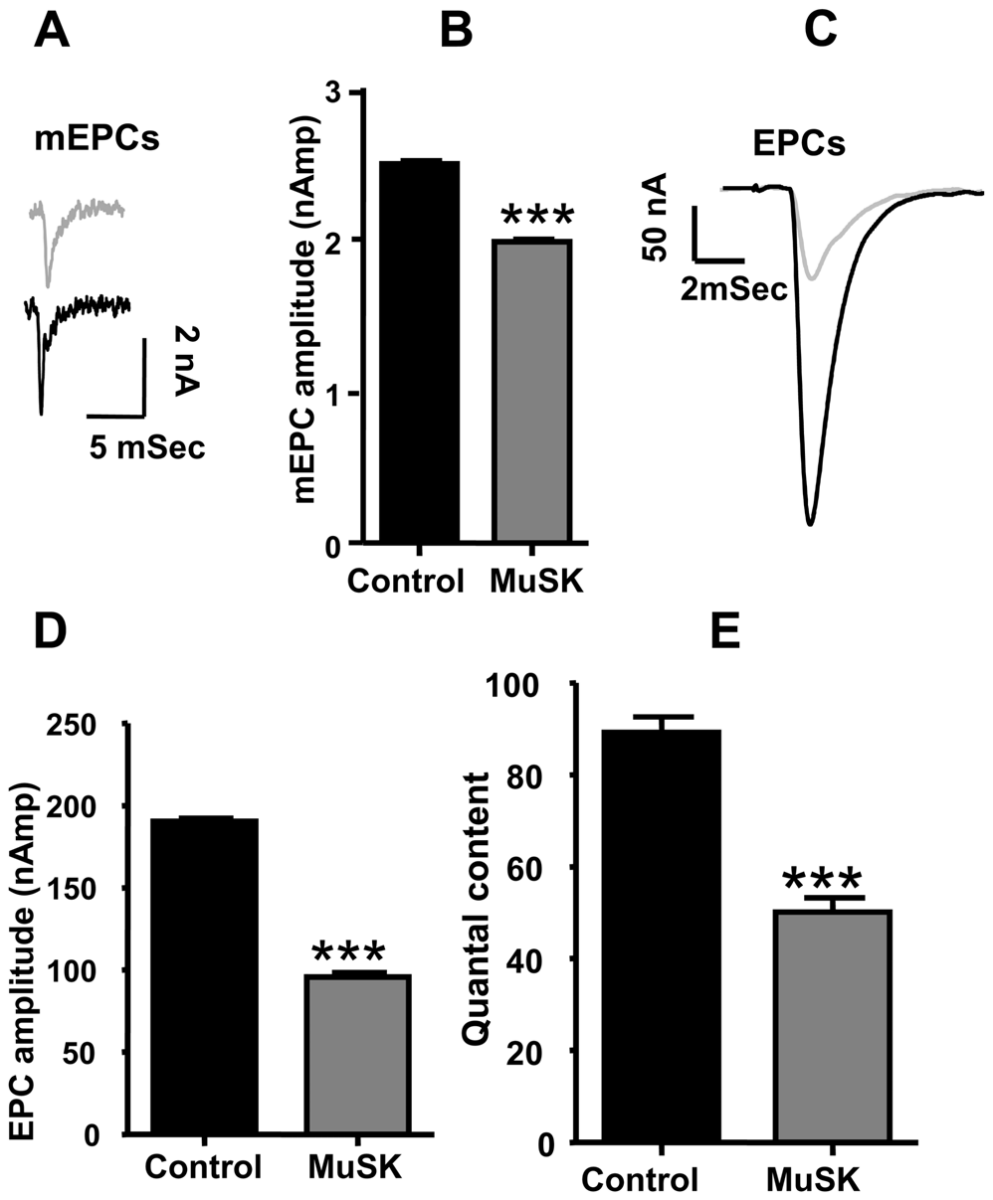
Neurotransmitter release is reduced for respiratory muscles of MuSK-MG-affected mice. (A) Representative mEPCs (A) and mean mEPC amplitude (B) of *Triangularis sterni* (TS) nerve-muscle preparations removed from control (black trace) and MuSK-MG-affected (grey trace) mice. Representative EPCs (C), mean EPC amplitude (D), and mean quantal content (E) of *Triangularis sterni* (TS) nerve-muscle preparations removed from control (black trace) and MuSK-MG-affected (grey trace) mice. Data bars for B, D, and E are mean + SEM of 40 endplates, in 8 MuSK_MG affected mice and for 41 endplates in 12 vehicle injected control mice; *** denotes P<0.0001.

**Figure 3:**
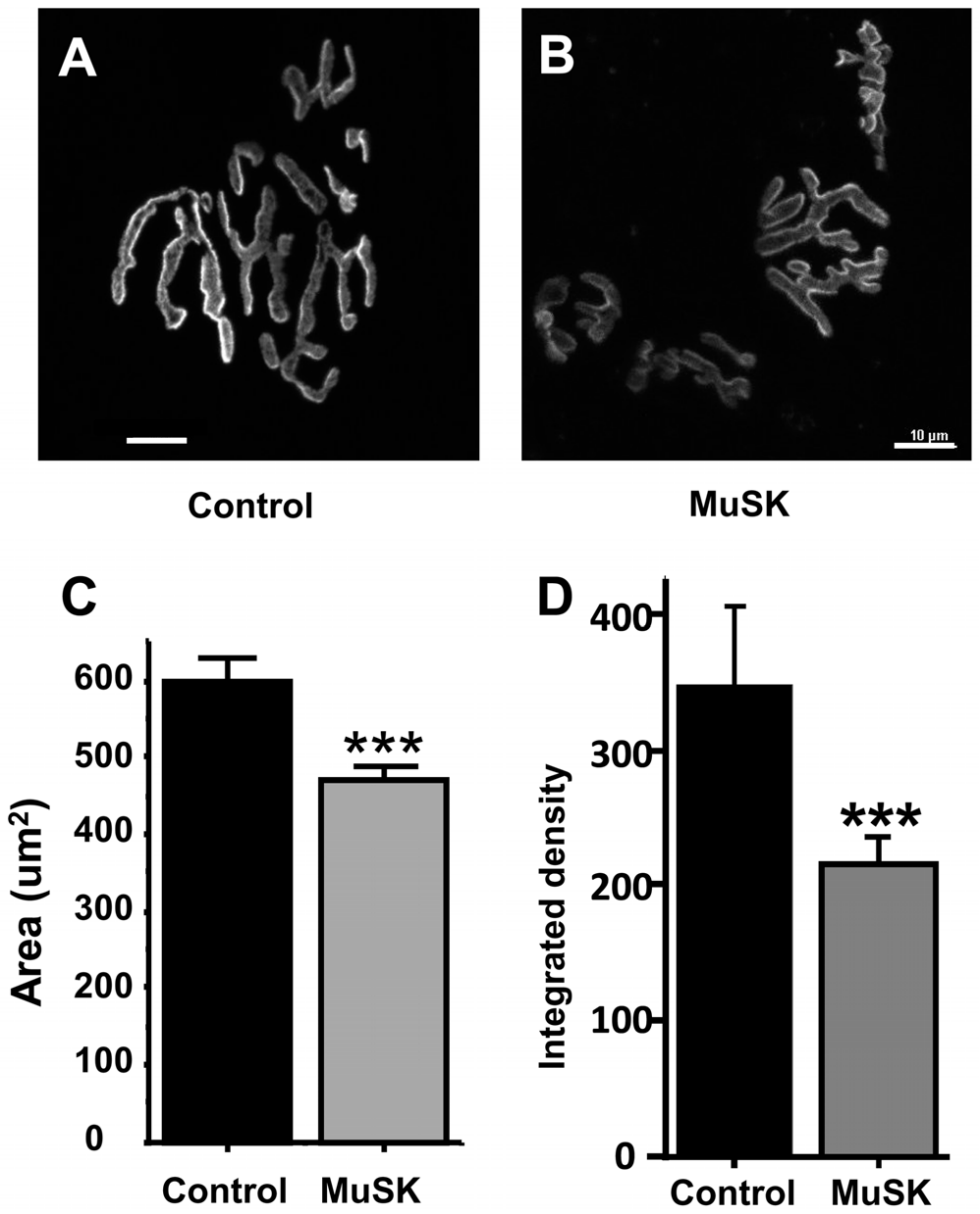
Endplate AChR density declines in MuSK-MG-affected mice. (A) & (B) are representative images of rhodamine α-bungarotoxin-stained endplates in the TS muscle isolated from a control or a MuSK-MG-affected mouse. The mean endplate areas (C) and integrated density (D) of vehicle injected control (n = 59 endplates, 5 mice) or MuSK-MG-affected (n = 32 endplates, 4 mice) mice, stained with rhodamine-labeled α-bungarotoxin is significantly reduced. Calibration bars represent 10 µm; data points are shown as the mean + SEM; *** denotes P<0.0001.

**Figure 4:**
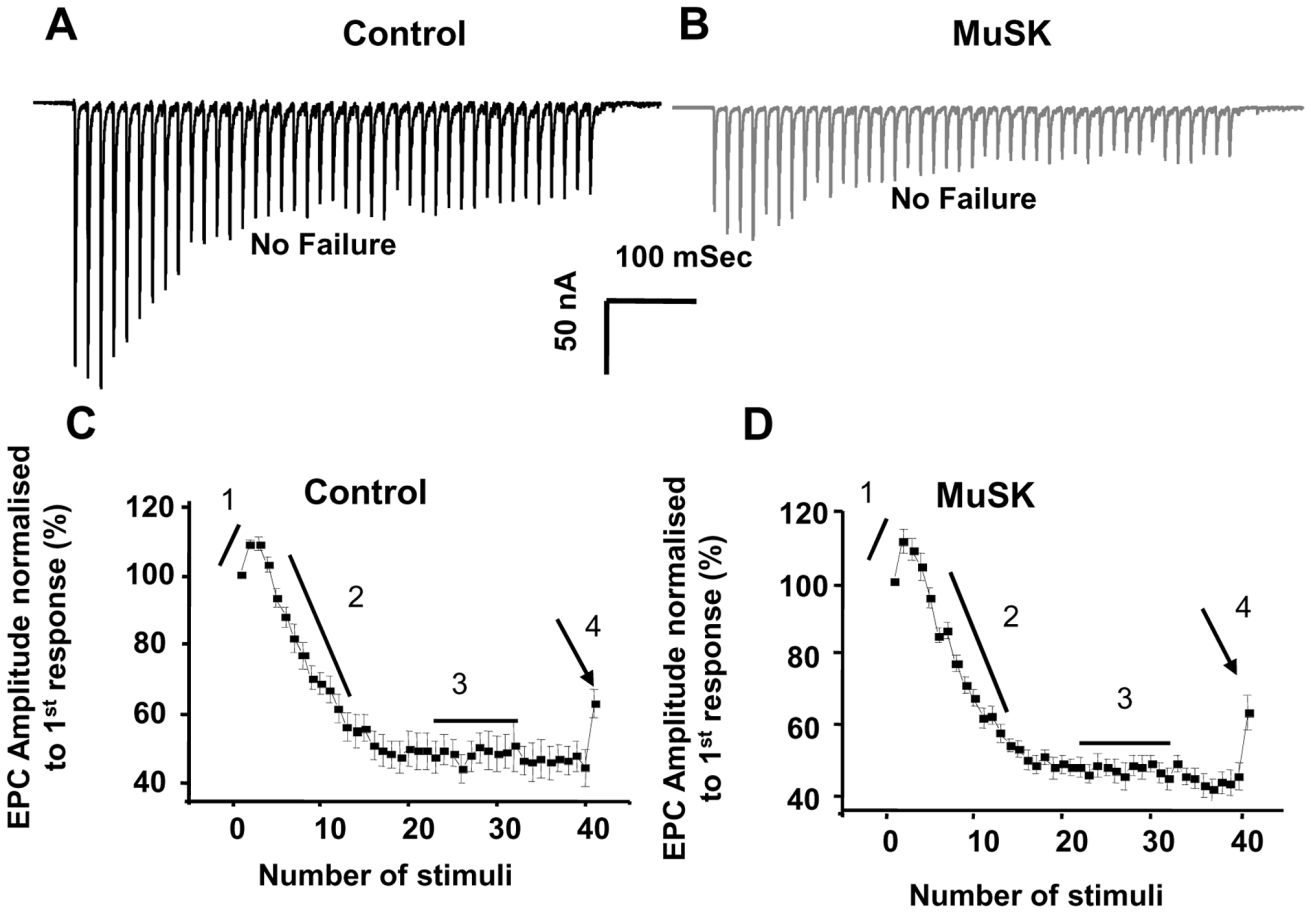
Kinetics of neurotransmitter release are qualitatively normal for non-failing NMJs of MuSK-MG-affected mice. Representative EPCs that do not fail in response to 50 Hz stimulus trains (40 stimuli) applied to TS preparations removed from control (A) and MuSK-MG-affected (B) mice. (C and D) EPC amplitudes were normalized to the first EPC in 50-Hz trains to demonstrate potentiation (1) and depression (2) of neurotransmitter release to a steady-state level (3). At 1 second after the end of the stimulus train (arrow), a challenge stimulus tested for recovery of neurotransmitter release (4). Data points are shown as the mean ± SEM.

**Figure 5:**
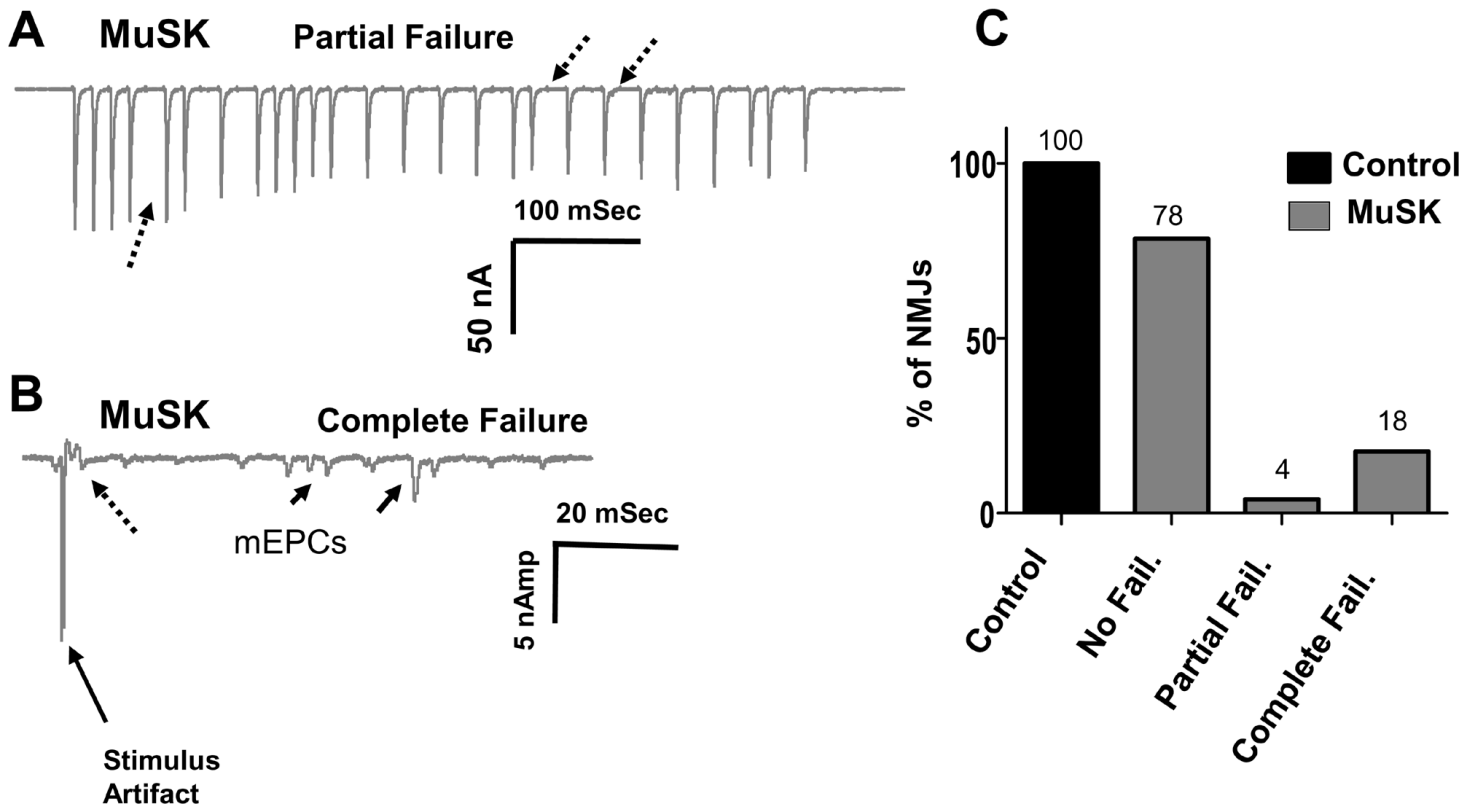
Partial and Complete failure of EPCs for TS preparations removed from MuSK-MG-affected mice. (A) Representative response of a NMJ showing partial failure of EPCs in response to a 50 Hz train. (B) Representative response of a “silent” NMJ having mEPCs (solid arrows), but complete failure of EPCs (broken arrow). (C) Bar graph showing the distribution of NMJs having no EPC failures, partial EPC failures, or complete EPC failures. EPCs were acquired from a total of 136 NMJs in 11 control and 176 NMJs in 14 MuSK-MG-affected mice.

Functional and morphological properties of another respiratory muscle, the TS, were studied in detail. The mean mEPC amplitude decreased from the control value of 2.5±0.02 to 2.0±0.01 nAmp for MuSK-MG-affected mice (Figure 2 A and B). This can be attributed to the decline of endplate AChR density (Figure 3 A to D). Likewise, the decline of endplate AChR density can contribute to the significant decrease of mean EPC amplitude (Figure 2 C and D). However, the ratio of mean EPC to mean mEPC amplitudes suggests that quantal content was significantly less for 78% of MuSK-MG-affected NMJs stimulated at 2 Hz for 1 min (Figure 2E). Similarly, the steady-state EPC amplitude was significantly less for these NMJs subjected to a train of 40 stimuli at 50 Hz. In contrast, transmitter release at the higher frequency of nerve stimulation remained qualitatively normal. That is, bursts of 50-Hz stimulus trains produced EPCs whose amplitude underwent initial potentiation followed by depression to the steady-state level: a challenge stimulus delivered at 1 Sec after the 50-Hz train revealed normal recovery of the release process (Figure 4 A to D). This pattern of high-frequency neuromuscular transmission was strikingly different for the remaining 22% of MuSK-MG-affected NMJs. That is, 4% and 18% of MuSK-MG-affected NMJs showed partial or complete EPC failure, respectively, in response to 50-Hz stimulus trains (Figure 5 A to C). It is important to note that mEPCs were detected at all NMJs with complete EPC failure and that failure was never observed for 136 NMJs in 11 control TS preparations. Subsequent experiments were performed to discover the basis for the reduced quantal content and high frequency EPC failure for MuSK-MG-affected mice.

### Reduced neurotransmitter release results from altered active zones and synaptic vesicles in MuSK-MG-affected mice

**Figure 6:**
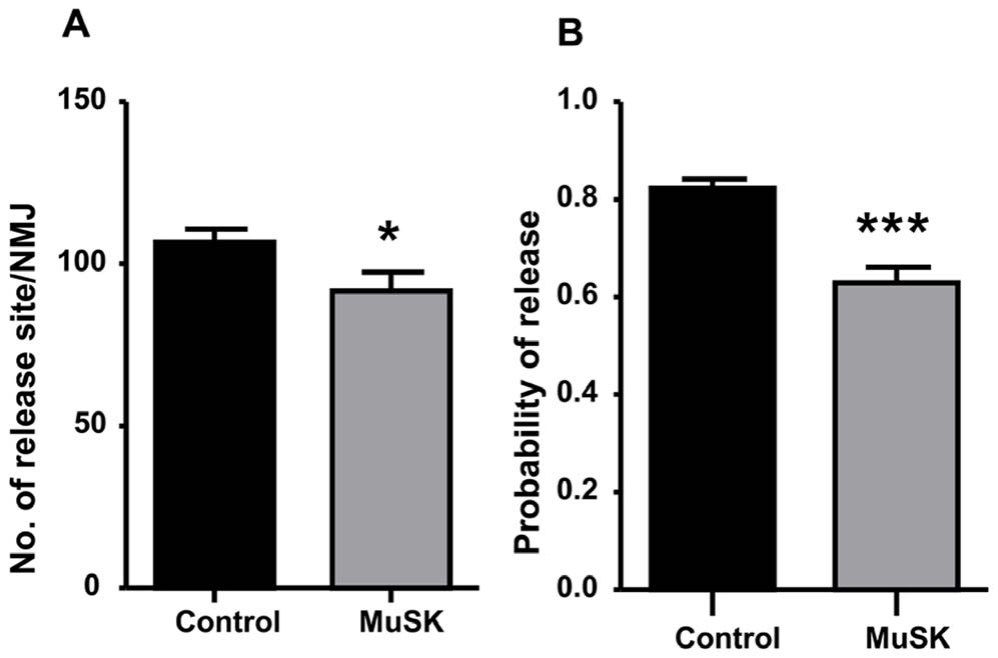
Number of vesicle release sites and probability of vesicle release are reduced for MuSK-MG-affected TS preparations. Equations 1 and 2 were used to calculate the number of vesicle release sites (A) and the probability of neurotransmitter release (B), respectively. Bars are shown as the mean + SEM for control (34 NMJs, 12 mice) and MuSK-MG-affected (31 NMJs, 8 mice) preparations; * and *** denote P<0.05 and P<0.0001, respectively.

Since quantal content is determined by the number of quanta release sites (N) and the probability of release (P), we calculated N and P from mEPC and EPC amplitude variability (equations 1 and 2). Figures 6 A and B show that both N and P of MuSK-MG-affected TS muscles were significantly reduced compared to control. Quantification of motor-nerve ending active-zone-associated proteins like piccolo or bassoon was not possible due to non-specificity of available antibodies. However, distribution of another active-zone protein, ELKS, on motor-nerve endings in the TS of MuSK-MG-affected mice was qualitatively abnormal (Figure S2).

**Figure 7:**
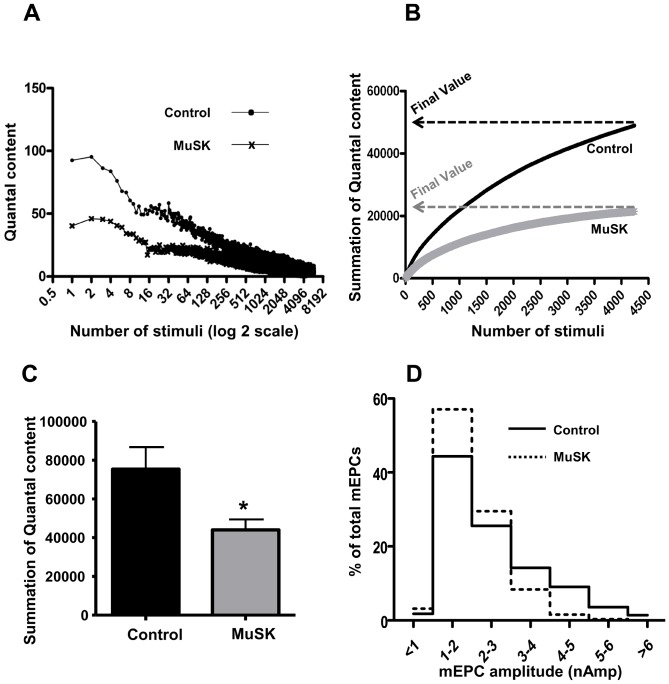
Reduced size of the functional vesicle pool within motor-nerve terminals contributes to lowered quantal content of MuSK-MG-affected TS preparations. (A) Representative time course of quantal content for EPCs in control or MuSK-MG-affected TS muscles that were stimulated at 50 Hz; X axis is the stimulus number (log 2 scale). (B) Representative graph showing the summation of quantal content during the 2 minute, 50 Hz train. The Y axis value is the summation of all quantal contents prior to the EPC number plotted on the X axis. The final value, or Y axis endpoint, of each curve is the total number of vesicles released during the 2 minute, 50 Hz stimulus train. (C) The total number of quanta released during the 2 minute, 50 Hz stimulus train is significantly (* P<0.05) less for MuSK-MG-affected (8 NMJs, 4 mice) muscles than that for vehicle-injected control muscles (7 NMJs, 3 mice). (D) Distribution of mEPC amplitudes as a percent of the total mEPCs used to calculate the mean amplitude shown in (Figure 2B). While the modal value remains equivalent to that of control, reduced numbers of mEPCs larger than 2–3 nA accounts for the decline of mean mEPC of MuSK-MG-affected TS preparations.

Since a decrease of the pool size of releasable vesicles would reduce neurotransmitter release, we recorded the decline of EPC quantal content during 2 mins of 50-Hz stimulation. Figure 7A plots quantal content of each EPC in the 2-min train, as a function of the stimulus number. Although quantal content is initially less for the MuSK-MG-affected TS preparations, both experimental and control values declined to a similar value. Figure 7B plots the cumulative number of quanta released during the 2-min train. The final Y-axis cumulative value for the entire train of EPCs is an estimate of the number of vesicles in the releasable pool. The final value for cumulative quantal estimate (Figure 7C) of MuSK-MG-affected muscles (44080±5340) was significantly less than control (75390±11380). An additional indication of abnormality in motor-nerve terminal processing of quanta is the decline of large amplitude mEPCs at MuSK-MG-affected NMJs. That is, while the modal amplitude (1–2 nAmp) was equivalent to control, the occurrence of mEPC amplitudes greater than 4 nAmp was markedly reduced for MuSK-MG-affected TS preparations (Figure 7D).

### Decreased nerve-terminal excitability causes partial EPC failures at NMJs in MuSK-MG-affected mice

**Figure 8:**
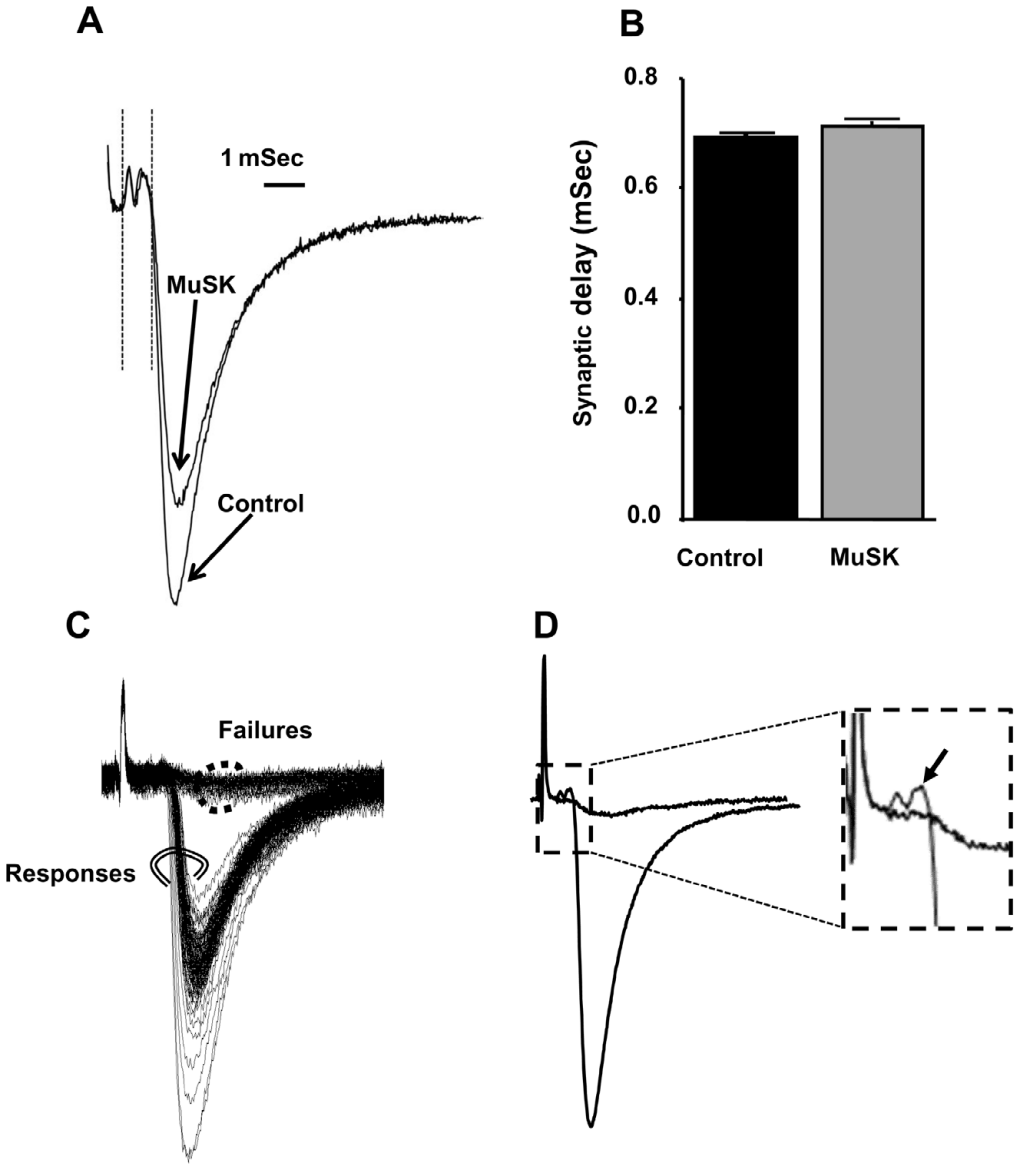
Reduced motor-nerve terminal excitability, but not prolonged synaptic delay, is responsible for high-frequency EPC failures of MuSK-MG-affected NMJs. (A) Representative extracellular recordings at NMJ of TS preparations isolated from control and MuSK-MG-affected mice lacking EPC failures. Each trace is the average of 120 recordings acquired at 50 Hz. Synaptic delay was measured as the time from the beginning of the nerve terminal current to the time at which the EPC amplitude was 10% of its maximum value, as indicated by the vertical lines following the stimulus artifact. (B) Synaptic delay is equivalent for control (70 NMJs, 4 mice) and MuSK-MG-affected (60 NMJs, 4 mice) muscles. (C) Series of 120 extracellular recordings made at an endplate of a MuSK-MG-affected NMJ, which exhibited partial failure in response to 50 Hz nerve stimulation. Double circled records clearly possessed EPCs, while dot circled records were failures of EPC initiation. (D) Average of double-circled and dot circled records of panel C show that failures of EPC initiation resulted from lack of nerve impulse entry into the nerve terminal; the boxed portion of the averaged records is amplified in the inset with an arrow indicating nerve terminal current in association with the EPC.

To explore the cause(s) of EPC failure, we next recorded nerve-terminal currents. Extracellular recordings from MuSK-MG-affected TS preparations that did not develop EPC failures during 50-Hz stimulus trains revealed that the nerve-terminal current preceding EPCs was qualitatively normal and the synaptic delay was equivalent to the control value of 0.7 mSec (Figure 8 A and B). In contrast, EPC failures for MuSK-MG-affected NMJs were associated with failure of action potential entry into the motor-nerve terminal (Figure 8 C and D). Within any MuSK-MG-affected TS muscle, NMJs with partial or complete EPC failures were adjacent to synapses with reduced quantal content, but without EPC failure. NMJs with failures (silent NMJs) always produced mEPCs [59]. Importantly, control NMJs never exhibited EPC failure.

### Morphological changes contribute to functional decline of motor-nerve terminals at NMJs in MuSK-MG-affected mice

**Figure 9:**
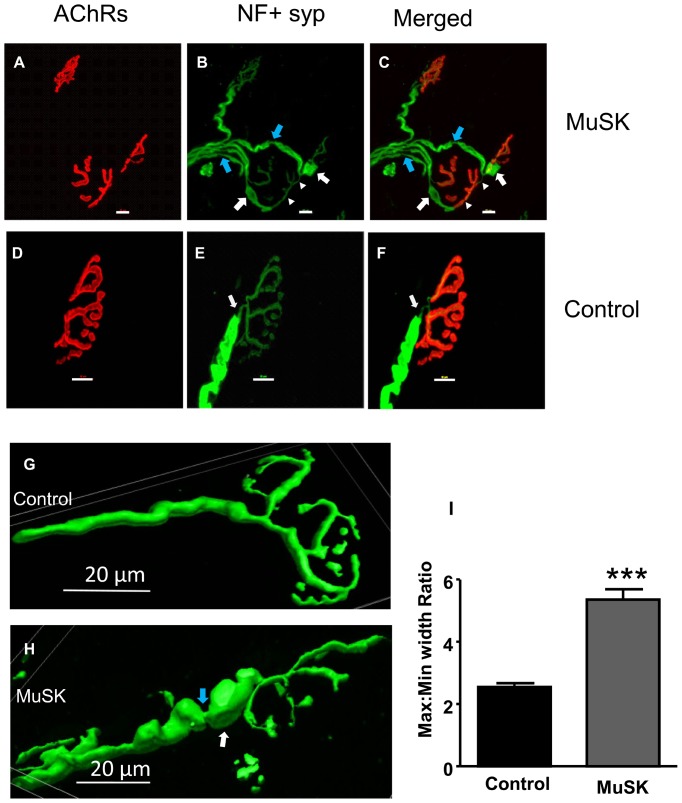
MuSK-MG-affected TS preparations have altered motor-nerve structure that may contribute to EPC failure. Maximum intensity projection images obtained from confocal optical slices of NMJs in the TS of MuSK-MG-affected (A-C) and control (D-F) mice. The AChR-enriched endplate area is stained red, while motor nerves are stained green. Morphological abnormalities observed for NMJs of MuSK-MG-affected mice include: double innervation (white arrow heads), neurofilament positive swellings (white arrows), and axonal thinning (blue arrows) in B and C. Representative 3D-reconstruction images derived from confocal optical slices of control (G) and MuSK-MG-affected (H) nerve terminals. (I) Graph showing mean (+ SEM) values of maximum to minimum motor-nerve terminal diameter within 25 µm of branch point in control and MuSK-MG-affected TS preparations; P<0.0001. Calibration bars represent 20 µm.

To detect morphological alterations that could compromise neuromuscular transmission, we examined motor nerves labeled with antibodies to neurofilament or synaptophysin. Remodeling of TS nerves in MuSK-MG-affected mice was detected as preterminal branching along with polyinnervation of muscle endplates (Figure 9 B and C, white arrowheads), as well as regions of axonal swelling and thinning (white and blue arrows, respectively, in Figures 9, C, G, and H). Measurement of the ratio of maximum to minimum axon diameter within 25 µm of the nerve-terminal branch point revealed a significant increase for MuSK-MG-affected TS preparations (Figure 9 I).

### Reduced EPC decay rate for MuSK-MG-affected mice is due to decline in AChE activity

**Figure 10:**
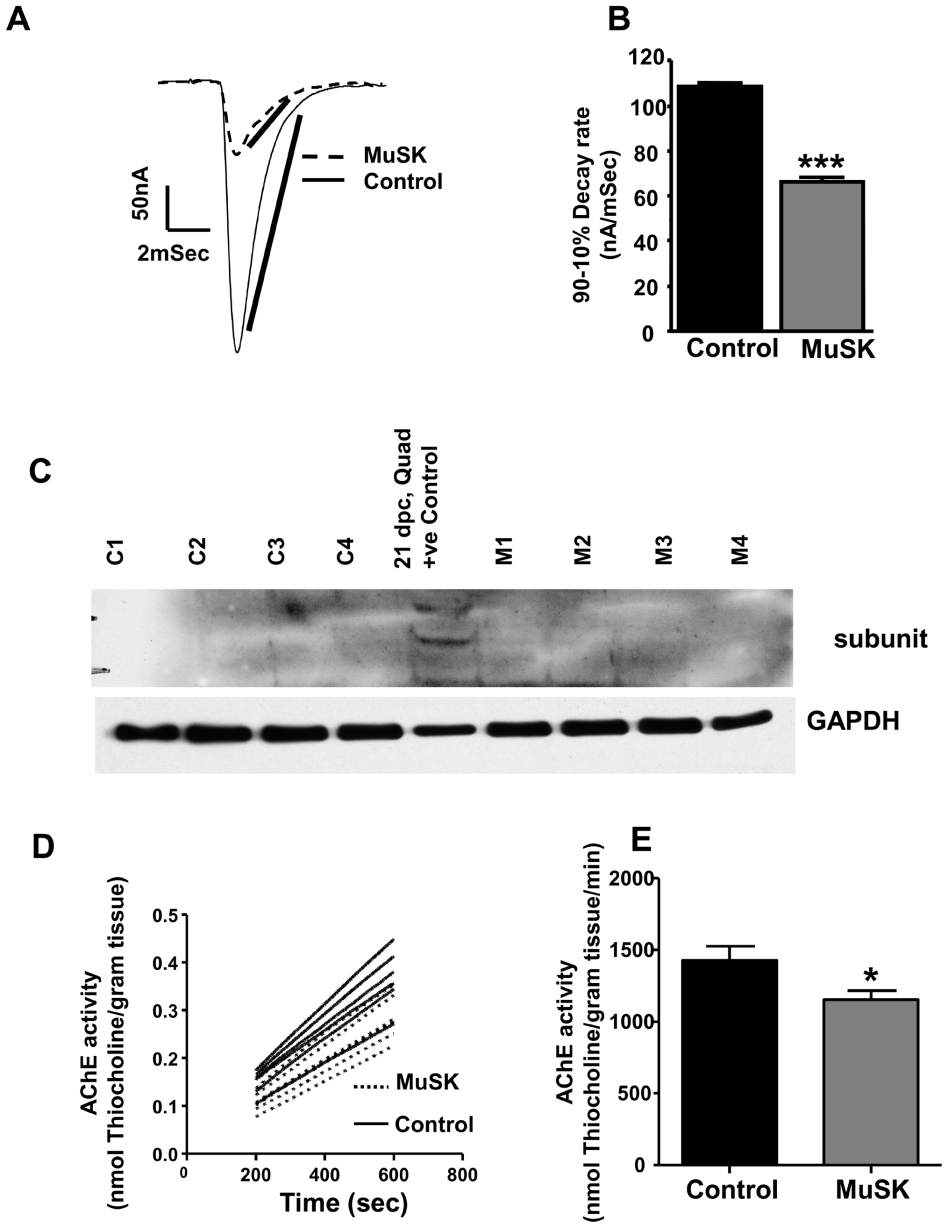
Reduced AChE activity but not reduced embryonic AChR expression accounts for the prolongation decay of EPCs in MuSK-MG-affected muscles. (A) Estimation of 90% to 10% decay rate for EPCs of TS muscles of vehicle injected Control (solid trace) and MuSK-MG-affected (dotted trace) mice. (B) EPC 90–10% decay rate for MuSK-MG-affected mice (2366 EPCs, 40 endplates, 8 mice) is significantly (P<0.0001) less than that for vehicle injected control mice (2128 EPCs, 41 endplates, 12 mice). (C) Western blot showing no expression of fetal AChR γ subunit in diaphragm muscles of 4 control (C1-C4) and 4 MuSK-MG-affected (M1-M4) mice. Quadriceps muscle of embryonic rat (ED21) served as a positive control (+ve Control). (D) Rate of thiocholine production in homogenates of hemidiaphragm muscles of vehicle-injected control (solid lines) and MuSK-MG-affected (dotted lines) mice indicates AChE activity. (E) AChE activity of MuSK-MG-affected mice (n = 7) is significantly (* denotes P<0.05) less than that of vehicle-injected control mice (n = 6).

**Figure 11:**
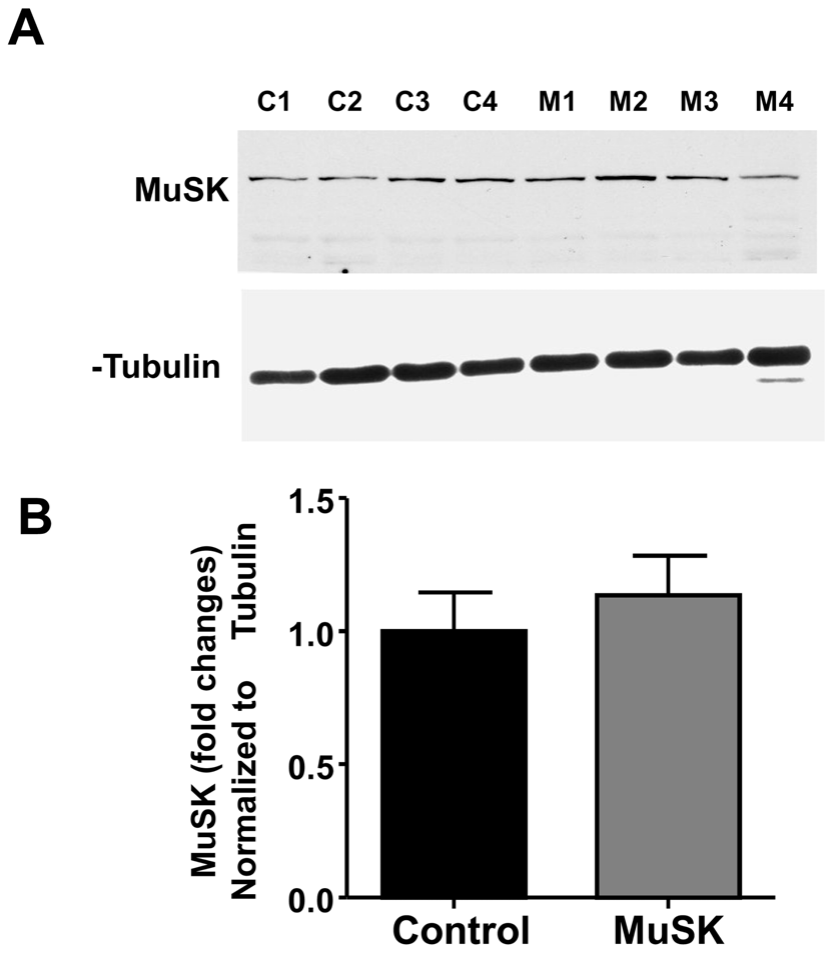
Expression of MuSK is equivalent for vehicle-injected control and MuSK-MG-affected mice. (A) Western blots showing MuSK protein in diaphragm muscle of control (C1-C4) and MuSK-MG-affected (M1-M4) mice; α-tubulin was used as the loading control. (B) Graph showing the mean ± SEM of changes in MuSK protein expression, normalized to α-tubulin expression for MuSK-MG-affected and control muscles.

In addition to presynaptic abnormalities, NMJs of MuSK-MG-affected mice produced EPCs whose 90% to 10% decay rate was significantly reduced to 66.4±1.0 from the control value of 109.0±0.8 nAmp/mSec (Figure 10 A and B). The decline of EPC decay rate could arise from either expression of the embryonic AChR or loss of acetylcholinesterase (AChE). The former possibility is excluded since MuSK-MG-affected muscles did not express the AChR γ subunit (Figure 10 C). In contrast, AChE enzymatic activity significantly declined from the control value of 1427±100 to 1155±62 nMol thiocholine/gram tissue/min for MuSK-MG-affected muscles (Figure 10 D and E). Since, MuSK regulates AChE at the NMJ, we measured protein levels and found that MuSK expression was equivalent for diaphragm muscles isolated from control and MuSK-MG-affected mice (Figure 11 A and B).

## Discussion

Animal models facilitate understanding of the pathophysiology of MuSK-MG. In our study, all MuSK-immunized mice produced anti-MuSK antibodies. Since the overall decline of rotarod performance, grip strength, and pulmonary function suggest generalized muscle weakness, it is possible that the 40% of MuSK-immunized mice whose diaphragm muscle was weakened may be a subset of mice that developed MG. We studied this subset of immunized mice in detail because 60% to 70% of MuSK-MG patients suffer respiratory weakness [16],[56],[57]. Significantly, the data of Figure S1 A and B show that MuSK-immunized mice without weak diaphragm muscles had TS muscles with normal quantal content. Furthermore, MuSK-MG weakens variable muscle groups [9]. Therefore, it is not surprising that 60% of MuSK immunized mice did not exhibit diaphragm muscle weakness.

It should be noted that we immunized the mice with rat MuSK ectodomain (residues 22-212) expressed in and purified from *E. coli*, which is shorter than that utilized in prior studies of active immunization MuSK-MG models [32],[33],[43]. The antigenicity of this shorter, bacterial-derived recombinant MuSK peptide may be less than for the full-length or longer fragments of the protein produced in mammalian cells. Ulusoy et al. also immunized mice with the ectodomain of human MuSK (residues 1-463) expressed and isolated from *P. pastoris* [60]. They observed that although mice immunized with 10 or 30 µg of this recombinant MuSK ectodomain produced comparable levels of antibody, MG symptoms were less frequent for mice receiving the lower dose. Finally, besides variable antibody titers, the differences in disease outcomes observed between MuSK-MG models may be attributed to differences in responses to passive immunization with human anti-MuSK-MG antibodies versus active immunization with recombinant MuSK ectodomains.

MuSK maintains endplate AChR density [21]–[23],[26] [61]. This determinant of the high safety factor for neuromuscular transmission declines for all models of MuSK-MG [31]–[34],[49] [62], and is likely due to suppression of MuSK-mediated pathways [62]. Our measurements of endplate labeling with bungarotoxin and mean mEPC amplitude support the work of others showing that endplate AChR density declines in experimental models of MuSK-MG. However, current literature suggests that endplate AChR density does not decline for a small number of weakened muscle biopsies obtained from MuSK-MG patients [30],[37]. In addition, Viegas et al. [33] observed that endplate AChR decline was equivalent for weak and non-weak muscles of MuSK-immunized mice. Thus, the role of endplate AChR decline in muscle weakness during MuSK-MG is uncertain. Furthermore, blockage of over 80% of AChRs is required to decrease the efficacy of neuromuscular transmission [63],[64]. Therefore, the approximate 20% decline of AChR density is, at best, a partial contributor to the reduced safety factor for neuromuscular transmission in the model of MuSK-MG that we study.

Animal models have revealed other abnormalities of the NMJ during MuSK-MG [31]–[34],[47]. Mori et al. [32] observed a decline of quantal content, while others observed that quantal content remains normal for MuSK-MG mice [33],[34],[49], although these latter studies report other abnormalities of nerve function. For example, Klooster et al. reported “…exaggerated depression of presynaptic ACh release during high rate activity” [34], while Viegas et al. [33], Klooster et al. [34], as well as Morsch et al. [65] did not detect a compensatory increase of quantal content, which occurs whenever AChR density declines [41],[42]. The lack of a compensatory increase in neurotransmitter release suggests that the decline of endplate AChR density during experimental MuSK-MG does not reach a threshold for activation of synapse homeostasis [39],[40].

Alternatively, the retrograde signaling pathway from muscle to nerve [45],[66] may be disrupted or motor nerves may be unresponsive to MuSK-Lrp4-mediated retrograde signals in the MuSK-MG models used by Viegas et al. [33] and Klooster et al. [34]. In contrast to all of these animal studies, Niks et al. [38] reported low levels of presynaptic acetylcholine release for a muscle biopsy obtained from a MuSK-MG patient. In light of the AChR dispersing action of ACh [67],[68], the decrease of neurotransmitter that Niks et al. reported may help sustain endplate AChR clusters. Overall, these conflicting observations stimulated us to further examine motor-nerve function as well as morphology during the active immunization model of MuSK-MG.

We observed three forms of motor-nerve dysfunction. The majority of MuSK-MG-affected motor-nerve endings had reduced EPC quantal content. Evaluation of mEPC and EPC amplitudes indicated a significant decline of N and P. These alterations imply a decline of functional vesicle release sites and action potential stimulated Ca^2+^ entry into motor nerve endings, respectively. Staining for the active zone protein ELKS [69],[70] suggested a dispersion of this active zone protein in MuSK-MG-affected motor-nerve endings. The decrease of quantal content was not associated with changes in nerve-terminal currents and synaptic delay or the potentiation, depression, and recovery of vesicle release seen in response to 50-Hz stimulus trains. These observations suggest that action potential entry into and activation of exocytosis remains qualitatively normal for nerve terminals with significantly reduced quantal content due to decreased N and P. Furthermore, the majority of affected nerve terminals contained a smaller pool of releasable vesicles and a selective loss of larger quantal events (Figure 7). The latter observation provides an alternate explanation for the decline of the computed mean mEPC amplitude. Furthermore, the modal mEPC amplitude was equivalent for control and MuSK-MG-affected muscles. If a decline of AChR density alone decreased the mean mEPC amplitude, then the amplitude distribution would uniformly undergo a leftward shift. Because this did not occur, MuSK-MG-affected nerve terminals may selectively produce fewer vesicles containing larger ACh concentrations. Both decline of releasable vesicle pool size and selective loss of larger quanta suggest altered vesicle processing during MuSK-MG.

A smaller population of MuSK-MG-affected motor-nerve endings exhibited a more severe suppression of neuromuscular transmission, where EPCs intermittently or completely failed in response to 50-Hz stimulation. Extracellular recording suggested that EPC failures were due to blocked nerve-impulse invasion into the motor-nerve terminals. Morphological analyses revealed axonal sprouting as well as swelling and thinning of terminal axons. It is striking that similar functional and morphological abnormalities occur for NMJs in periaxin-null mice [71]. Therefore, altered axon morphology may contribute to EPC failure for MuSK-MG-affected NMJs, as previously suggested for mice with “motor endplate disease” [72].

Overall, our data suggest the following presynaptic alterations during MuSK-MG. Motor-nerve terminals are altered so that the processes maintaining N, P, and vesicle formation are suppressed. Morphological alterations might block action potential entry into the nerve terminal to cause EPC failure. In agreement with EPC failure, Klooster et al. [34] reported fade of tetanic tension for nerve-muscle preparations isolated from MuSK-MG animals. However, the relatively short stimulus trains that we applied did not produce significant tetanic fade. The sustained tetanic responses of MuSK-MG-affected diaphragm preparations may also be due to low occurrence of NMJs with partial EPC failure during the time course of our study. Further investigation is required to relate morphological and functional alterations of motor-nerve terminals in the active immunization model of MuSK-MG. In particular, subtle repositioning of pre- and postsynaptic components of the NMJ that decrease the efficacy of the neurotransmitter may precede the dramatic morphological changes that we report [73],[74].

MuSK regulates other NMJ proteins besides the AChR. For example, AChE deposition at the NMJ involves interactions of MuSK, ColQ, and the esterase enzyme [29],[75],[76]. MuSK-MG patient refractoriness to, as well as the worsening of symptoms during treatment with, anti-AChE drugs [12],[32] can be attributed to: 1) Low AChE activity [30],[32]; 2) MG-induced decline of the safety factor for neuromuscular transmission [44],[77],[78]; or 3) effect of ACh dispersal on AChR clusters [49],[65]. The dispersal effect of ACh on AChR could be unopposed, if antibodies prevent MuSK activation by agrin [67],[68].

Mori et al [32] attributed endplate potential prolongation during experimental MuSK-MG to reduced NMJ levels of AChE. Prolonged opening of AChRs would thereby enable excessive Ca^2+^ accumulation at, and subsequent degeneration of, the motor endplate [79]. Consequently, ACh mediation of functional neuromuscular transmission may be in a delicate balance with ACh suppression of AChR clusters in myasthenias, where pathology alters MuSK, or functionally related proteins. Manipulation of this balance may be critical to successful, long-term therapy of MuSK-MG patients. In addition to anti-AChE drugs, activators of neurotransmitter release could alter this balance. In fact, acute administration of 3,4-diaminopyridine (DAP) improves neuromuscular transmission in a model of MuSK-MG [43]. This effect of DAP is consistent with the decline of P, which we report. However, it is essential to evaluate the long-term consequences of DAP administration during MuSK-MG. In the long term, an increase of ACh release could worsen weakness in MuSK-MG patients as occurs with anti-AChE [18]. It is relevant to note that pyridostigmine disperses endplate AChRs in mice injected with human MuSK antibodies, while DAP did not have this effect even though it increased quantal content [49]. Therefore, current knowledge suggests that the neurotransmitter release process is a promising therapeutic target for MuSK-MG that deserves thorough study.

Activity is critical to maintenance of the peripheral neuromuscular apparatus. For example, AChE insertion at the NMJ relies upon muscle activity [80]. Inactivity induces embryonic AChR expression, which would prolong neurally-evoked endplate signals [81],[82]. However, γ subunit re-expression did not occur in our mouse model, suggesting that the level of MuSK-MG-induced muscle inactivity was not sufficient to activate γ subunit expression. Although the prolonged EPCs that we recorded most likely arose from a decline of AChE activity, MuSK-MG induced structural changes of the NMJ could also alter the efficacy of neurotransmitter action.

The predominant MuSK antibodies, IgG1 in mouse and IgG4 in patients, do not destroy the motor endplate through complement-mediated tissue destruction [32],[37],[58],[60]. In this regard, it is relevant that we did not detect a decline of total MuSK protein level in muscles of affected mice. Antibody-mediated disruption of MuSK function would account for the reduction of mEPC amplitude and endplate AChR density. However, we did not study the distribution and function of MuSK at the endplate, so we cannot exclude the possibility that MuSK antibodies altered synaptic localization and/or function of MuSK. Furthermore, Lrp4, which forms a complex with MuSK and acts as the endplate agrin receptor [83],[84], is critical to retrograde signaling at NMJs [66]. Therefore, antibody binding to select MuSK epitopes may alter MuSK-Lrp4-mediated retrograde signal(s) [45],[85].

Currently, the retrograde signaling cascade linking MuSK to nerve terminal function is unknown. However, NMJs of mice lacking all three forms of neural cell adhesion molecules (NCAM) resemble those that we describe for MuSK-MG-affected mice, where neuromuscular transmission fails during high frequency stimulation, vesicle cycling mechanisms develop poorly, and trafficking of active zones is altered [86]. Diffuse distribution of presynaptic proteins, such as P/Q-type Ca^2+^ channels, on motor-nerve endings of NCAM-null mice may decrease the probability of vesicular release [87]. NCAM may also act via myosin light chain kinase (MLCK) pathways [88]. These similarities between NCAM knockout and MuSK-MG-affected NMJs suggest signaling cascades that could be further studied during MuSK-MG.

### Conclusion

Results from our data provide novel insight into the pre-synaptic pathophysiology of MuSK-MG. Changes in vesicle dynamics, altered active zones, and presynaptic conductivity define functional abnormality in the motor-nerve terminals. These findings together with post-synaptic decline of AChRs are the factors contributing towards muscle weakness during MuSK-MG. Additional work is needed to elucidate the retrograde pathways linking MuSK to presynaptic function.

## Supporting Information

Figure S1 **Musk immunized mice with normal force of contraction of phrenic nerve diaphragm preparations also had normal quantal content of TS preparations.** (A) Each bar represents mean + SEM twitch tension for control, MuSK-MG-affected and not affected mice. Number of mice studied is ‘n’. (B) Each bar represents mean + SEM quantal content for control, MuSK immunized affected and not affected mice. Number of mice studied is ‘n’. *** denote P<0.0001.
(TIF)

Figure S2 **Immuohistochemical staining for ELKs suggests altered distribution of active zones during MuSK-MG.** (A and B) Confocal microscopic images of motor endplates in control and MuSK-MG affected TS preparations, stained for ELKs (Green) or AChR (Red).
(TIF)
