# Supplementary figures and images for: Altered Active Zones, Vesicle Pools, Nerve Terminal Conductivity, and Morphology during Experimental MuSK Myasthenia Gravis
Source: PLoS One. 2014 Dec 1;9(12):e110571. doi: 10.1371/journal.pone.0110571 (PMC4249869; doi:10.1371/journal.pone.0110571)

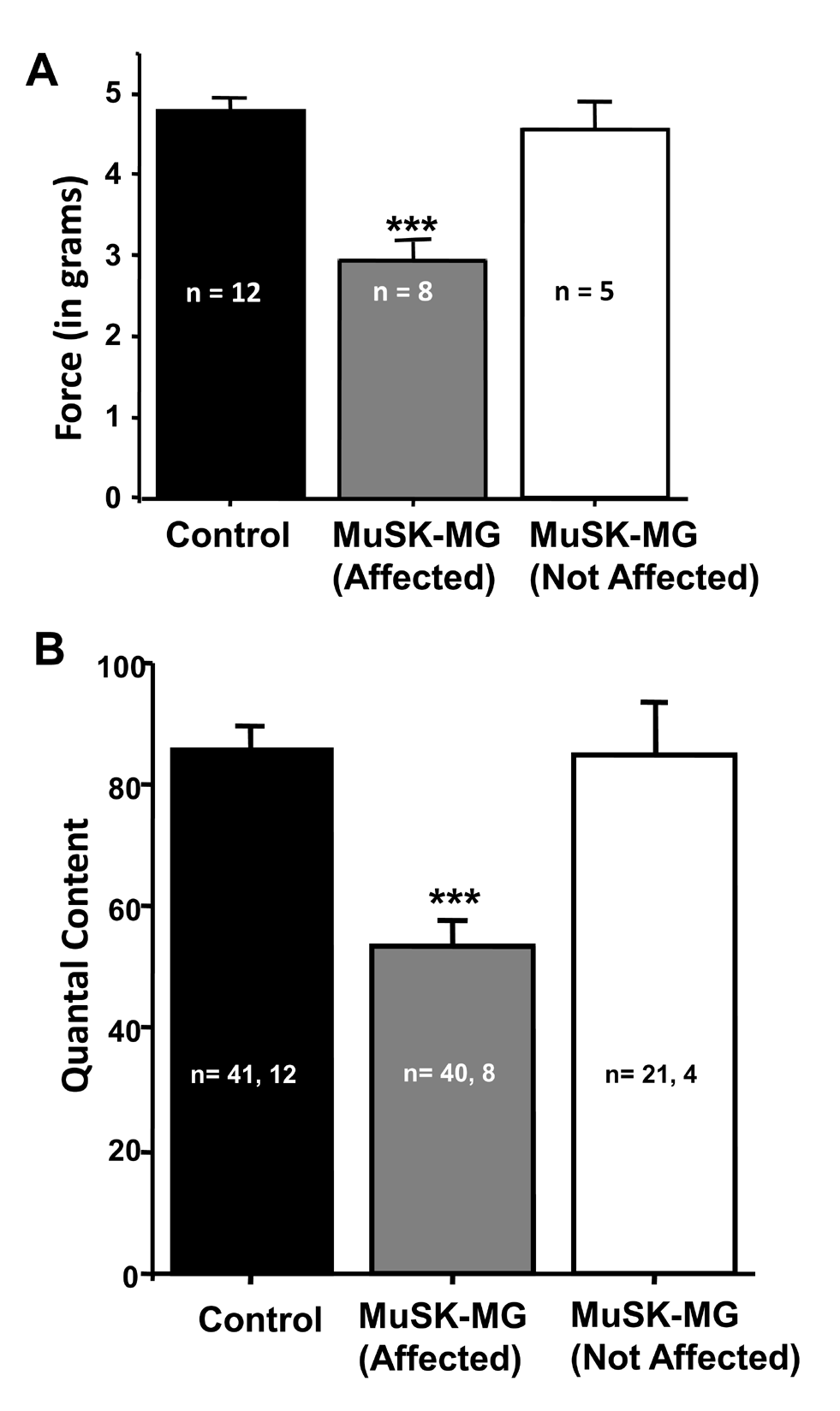

Supplement: Figure S1 — Musk immunized mice with normal force of contraction of phrenic nerve diaphragm preparations also had normal quantal content of TS preparations. (A) Each bar represents mean + SEM twitch tension for control, MuSK-MG-affected and not affected mice. Number of mice studied is ‘n’. (B) Each bar represents mean + SEM quantal content for control, MuSK immunized affected and not affected mice. Number of mice studied is ‘n’. *** denote P<0.0001. (TIF) [file pone.0110571.s001.tif]

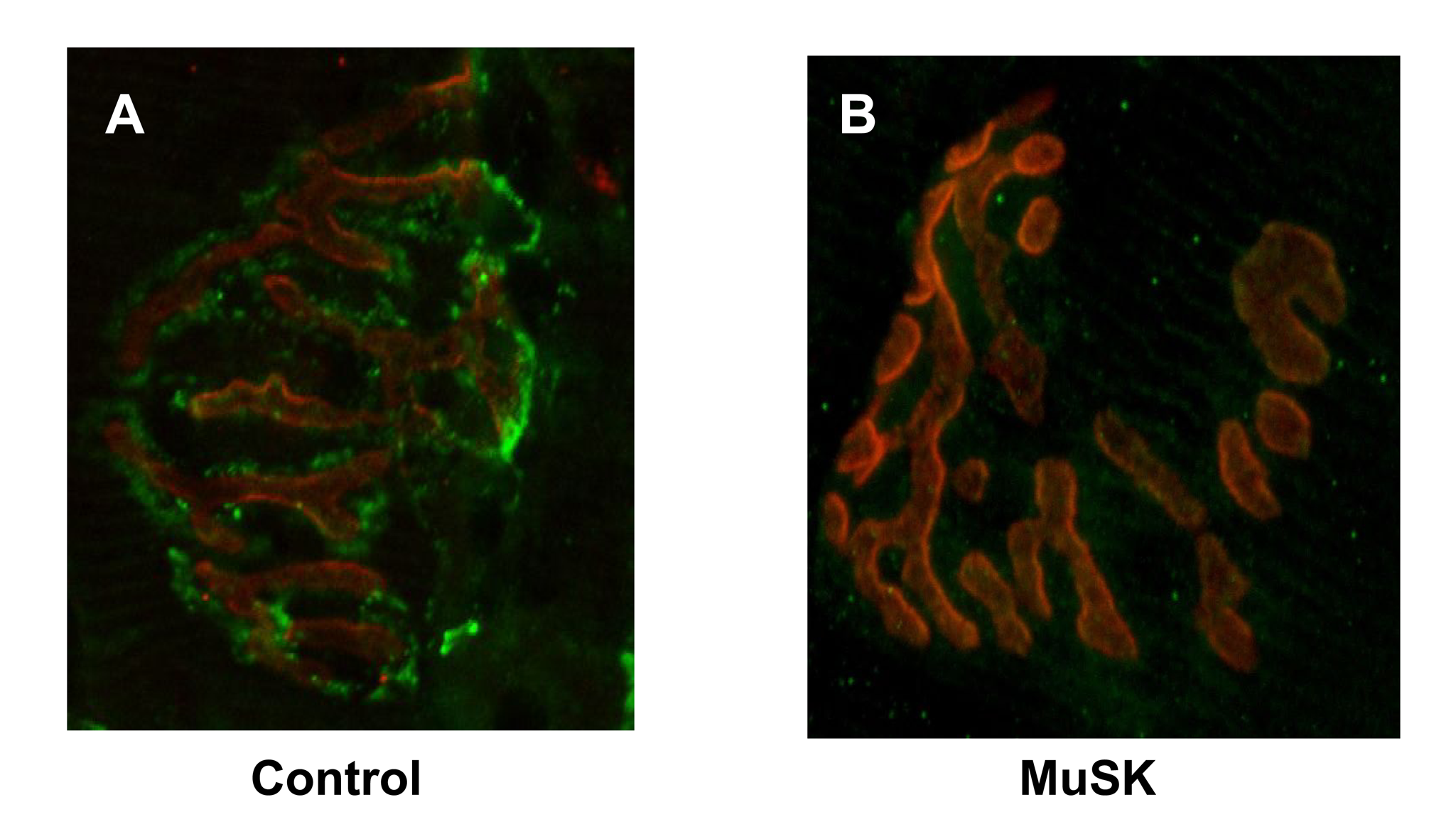

Supplement: Figure S2 — Immuohistochemical staining for ELKs suggests altered distribution of active zones during MuSK-MG. (A and B) Confocal microscopic images of motor endplates in control and MuSK-MG affected TS preparations, stained for ELKs (Green) or AChR (Red). (TIF) [file pone.0110571.s002.tif]
